# Risk factors for isolated atrial septal defect secundum morbidity

**DOI:** 10.1038/s41598-024-55446-2

**Published:** 2024-02-27

**Authors:** Gustaf Tanghöj, Estelle Naumburg

**Affiliations:** Unit of Pediatrics, Department of Clinical Sciences, Umeå University, Umeå, Sweden

**Keywords:** Paediatric research, Heart development

## Abstract

Atrial septal defect secundum is a common type of congenital heart defect and even more common among children born premature. The aim of this study was to assess premature birth as a potential associated risk factors for cardiac morbidity in children with isolated ASD II. In this retrospective national registry-based case–control study all children born in Sweden between 2010 and 2015 with an isolated ASD II diagnosis were included. Association between premature birth and cardiac morbidity in children with isolated ASD II was assessed by different outcomes-models using conditional logistic regression and adjustments were made for confounding factors. Overall, 11% of children with an isolated ASD II received treatment for heart failure. Down syndrome was the only independent risk factors for associated with cardiac morbidity in children with ASD II (OR = 2.25 (95%CI 1.25–4.07). Preterm birth in children was not associated with an increased risk of ASD II cardiac morbidity.

## Introduction

Secundum atrial septal defect (ASD II) is a common type of congenital heart defect (CHD). A birth prevalence of 164 in 100,000 live births have been reported and it is demonstrated to be even more common among children born premature^1–4^. Most children of ASD II remain asymptomatic during infancy and for smaller non-symptomatic ASD II a spontaneous closure is common^1,5^. Interventional ASD closure is recommended in asymptomatic children with a significant hemodynamic shunt at an age of 3–5 years^6,7^. ASD patients have a higher life long-term mortality than the general population, but the overall morbidity due to ASD in young children is less studied^7,8^. Symptoms like heart failure and in need of treatment, growth retardation, and frequent infections may indicate an earlier interventional closure^7^. Several studies have demonstrated that children born preterm have an increased risk of a spontaneous closure later compared with children born term^9,10^. Further, the atrial shunt may have negative impact on conditions like pulmonary hypertension^11,12^. A failure to wean off from respiratory support for children born preterm with an open ASD may and a benefit from early intervention has been shown^10–13^.

In Sweden, approximately 6% of all children are born before the 37th gestational week and thus are considered as premature^14^. Decreased left ventricular dimensions, increased left ventricular mass, increased right ventricular mass, and decreased right ventricular ejection fraction are present among preterm born children up to adolescence^15^. These changes may increase the risk of early heart failure due to an ASD II shunt, especially in combination with comorbidities like pulmonary diseases, which are associated with preterm birth^15–17^. We hypothesize that these myocardial alterations in children born preterm may increase the risk of cardiac morbidity. This cardiac impairment can be presented as an increased use of cardiac drug treatment or increased number of pediatric cardiac outpatient visits during infancy^10,12,18^.

The aim of this study was to assess premature birth as an independent risk factor for cardiac morbidity in children with isolated ASD II.

## Methods

All children born in Sweden between 2010 and 2015 and diagnosed with an isolated ASD II according to the European Paediatric Cardiac Code (EPCC) 05.04.02^19^, were retrieved from The Swedish Registry of Congenital Heart Disease (SWEDCON) and included in the study. Children diagnosed with persistent foramen ovale (PFO) (EPCC code 05.03.03) were not included.

The following information was retrieved from SWEDCON for all included patients with ASD II: First date of drug treatment of heart failure, dates and number of outpatient visits. Registration on medicines in SWEDCON is based clinical condition (the intention to treat) and information on type of drug (ATC-code) is missing. By individual linkage to the Swedish National Medical Birth Register (MBR) potential perinatal risk factors, demographic data, and gestational age at birth was retrieved. Retrieval was performed by the National Board of Health and Welfare and data were anonymized.

Gestational age at birth was stratified in accordance with the definition used by World Health Organization’s (WHO) and preterm birth was defined as birth prior to 37 gestational weeks^20^ .

All data are presented as means with standard deviation (std), median with interquartile range (iqr) or percentage (%), depending on distribution and fit. Student’s t-test (unpaired two-sided) was used for parametrically distributed variables, Pearson’s χ^2^ for categorical data, and Fisher’s exact test when cell values below five and Mann–Whitney U test for non-parametrically distributed variables. In this study, *p* values < 0.01 were considered significant.

Cardiac morbidity was assessed by two end-points and analyzed in separate analysis.

A. Morbidity defined by use of any kind of drug treatment for heart failure.
  Cases: Isolated ASD II with drug treatment for heart failure. Controls: Isolated ASD II without drug treatment for heart failure.
B. Morbidity defined as increased number of visits to cardiac outpatient clinic.
  Cases: Isolated ASD II with six or more visits to a pediatric cardiac outpatient clinic during a follow up period of 5 years. The increased number of visits was calculated by using the third interquartile range of number of visits among all included children with ASD II^21^. Controls: Isolated ASD II with five or fewer number of visits to a pediatric cardiac outpatient clinic.

Conditional logistic regression was performed to assess the association between cardiac morbidity in an isolated ASD II, as defined above, and having been born premature. Male gender and Down syndrome may influence the exposed risk factor (premature birth) as well as the outcome (cardiac morbidity). Thus, adjustments were made for these confounding factors. Potential mediators for morbidity in preterm born children such as persistent pulmonary hypertension (PPHN) and bronchopulmonary dysplasia (BPD), were not included as confounders.

The risk factor of preterm birth was defined in three different models. First, as all births prior to gestational age of 37 weeks. Second, defined by preterm subgroups as described by the WHO; born between 32 and 37 gestational weeks, born 28–31 gestational weeks, and born prior to 28 gestational weeks. Third, gestational age was used as a continual variable.

Maximum-likelihood estimates of odds ratio (OR) and 95% confidence interval (CI) were obtained. IBM SPSS Statistics software (version 27, IBM Corporation, New York, USA) was used.

This study was conducted in accordance with the ethical standards of the 1964 Helsinki Declaration with amendments and approved by the ethics committee of human research at Umeå University (Dnr 2017/86-31). The study is based on data retrieved from national registers. Informed consent is obtained from all subjects and/or their legal guardian(s) as they accepted to admit data to the register.

The IBM SPSS Statistics, Version 25 Software (IBM Corporation, New York, USA) was used.

## Results

### Demographics

This study included 762 children diagnosed with isolated ASD II. Among these, 81 (11%) children were prescribed a drug for heart failure, and 193 (26%) had more than five outpatient visits. The mean number of outpatient visits was 3.7 (± 3.1) and median age at the first visit was 64 days (IQR: 131 days). Further demographic data are presented in Table 1.

**Table 1:** Demographic and general information.

Total	N:762			
	Mean	Std	Frequency (n)	%
Number of OUTPATIENT visits (n)	4	± 30		
Weight at birth (g)	3118	± 898		
	Median	IQR		
Gestational age (w)	38	4		
Age at first visit (d)	64	131		
Age at use of first Heart failure drug (d)	104	307		
Born prematurely			176	23
Born 32 to 37 gestational weeks			110	14
Born 28 to 32 gestational weeks			33	4
Born before 28 gestational weeks			33	4
Very low birthweight			55	7
BPD			17	2
PPHN			19	3
DOWN SYNDROME			59	7

BPD, Bronchopulmonary dysplasia; D, days; G, grams; N, number; PPHN, Persistent pulmonary hypertension of the newborn; W, Weeks.

The potential mediators BPD (bronchopulmonary dysplasia), PPHN (persistent pulmonary hypertension of the newborn), and Down syndrome were evaluated. BPD was significantly more common among children born preterm than among term-born children. However, children with PPHN or Down syndrome were equally distributed among children born preterm and term born children (Table 2). No significant differences in number of outpatient visits (3.7 ± 3.0 std vs. 3.6 ± 3.1 std, *p* = 0.800) or age at first visit (111.0 days 172 iqr vs. 98.0 days 296.0 iqr, *p* = 0.824) were found between preterm born children and term children.

**Table 2:** Frequencies of comorbidities among preterm and term children.

	Preterm (n = 176)	Term (n = 767)	*p*-value
BPD	17 (10%)	0 (0%)	< 0.001
PPHN	8 (5%)	11 (2%)	0.047
DOWN SYNDROME	11 (6%)	39 (7%)	0.854

BPD, Bronchopulmonary dysplasia; PPHN, Persistent pulmonary hypertension.

Drugs for pulmonary hypertension were used in 12 children, among them seven (58%) were ex-premature and five (42%) were term children, *p* < 0.01.

### Risk factors

Children with drug treatment for heart failure treatment were more often diagnosed with PPHN than children without treatment (6% vs. 2% *p* = 0.03) and were younger at first visit (64.0 days 131.0 iqr vs. 109.5 days 278.0 iqr, *p* < 0.01) compared to children with no drug treatment for heart failure . Children with more than five outpatient visits were younger (67.0 days 161.0 iqr vs. 119 days 285.0 iqr, *p* < 0.01) at their first visit than children with fewer than five outpatient visits. Down syndrome was significantly more common among those with more than five outpatient visits (Table 3).

**Table 3:** Distribution of risk factors in each group.

Children with isolated ASD II AND…	…Heart failure drug TREATMENT			…More than five outpatient visits		
	Cases (n = 81)	Controls (n = 680)	*p*-Value	Cases (n = 196)	Controls (n = 549)	*p*-Value
Girl	47 (58%)	384 (56%)	0.779	111 (57%)	310 (57%)	0.968
Boy	34 (42%)	297 (44%)		85 (43%)	239 (43%)	
preterm	20 (25%)	156 (23%)	0.724	48 (25%)	128 (22%)	0.429
Born 32 to 37 gestational weeks	10 (12%)	100 (15%)	0.568	28 (14%)	77 (14%)	0.909
Born 28 to 32 gestational weeks	5 (6%)	28 (4%)	0.391	13 (7%)	19 (4%)	0.058
Born before 28 GESTATIONAL weeks	5 (6%)	28 (4%)	0.391	7 (4%)	24 (4%)	0.639
Very low birthweight	8 (10%)	47 (7%)	0.328	17 (9%)	36 (7%)	0.323
BPD	4 (5%)	13 (2%)	0.081	2 (1%)	15 (3%)	0.168
PPHN	5 (6%)	14 (2%)	0.025	5 (3%)	13 (2%)	0.886
Down SYNDROME	8 (10%)	22 (6%)	0.202	21 (11%)	28 (5%)	0.006
More than five outpatient visits	66 (81%)	130 (20%)	< 0.001	–	–	–
TREATMENT with heart failure drugs	–	–	–	66 (34%)	15 (3%)	< 0.001

BPD, Bronchopulmonary dysplasia; PPHN, Persistent pulmonary hypertension.

Children with drug treatment for heart failure had more outpatient visits (8.4 days ± 4.7 std vs. 3.1days ± 2.2 std, *p* < 0.01) than children without drug treatment. Further data on risk factors for drug treatment are provided in Table 3.

There was no independent risk factor for cardiac morbidity due to ASD II among children born preterm (Table 4). Down syndrome was associated with an increased risk of ASD II morbidity described as more than five outpatient visits (Table 4).

**Table 4:** Adjusted risk for heart failure drug treatment and risk factors for making more than five outpatient visits.

	Heart failure treatment		Risk factors for > five OUTPATIENT visits	
	OR	CI 95%	OR	CI 95%
Born preterm	1.10	0.64–1.88	1.17	0.79–1.72
Boy	1.08	0.67–1.72	1.04	0.74–1.44
Down SYNDROME	1.68	0.76–3.72	2.25	1.25–4.08
Term	ref		ref	
Born 32 to 37 gestational weeks	0.84	0.42–1.70	1.02	0.63–1.65
Born 28 to 32 gestational weeks	1.37	0.46–4.10	1.80	0.87–4.13
Born before 28 GESTATIONAL weeks	1.79	0.71–4.50	1.11	0.51–2.46
Boy	1.06	0.66–1.69	1.02	0.73–1.42
Down SYNDROME	1.79	0.80–3.99	2.37	1.28–4.22
Gestational weeks	0.97	0.91–1.02	0.98	0.94–1.03
Boy	1.06	0.66–1.70	1.03	0.74–1.44
Down SYNDROME	1.69	0.76–3.75	2.25	1.25–4.07

## Discussion

In this national case–control study we assessed the association between premature birth and risk of increased cardiac morbidity in children with secundum atrial septal defect (ASD II). Cardiac morbidity due to ASD II was not independently associated with premature birth, although persistent pulmonary hypertension and BPD was more common among children born preterm. Down syndrome was independently associated with increased morbidity due to ASD in our study.

Allover 11% of children with an isolated ASD II in this study, received treatment for heart failure, which is in line with previous findings^22,23^. Preterm birth, which includes several mediators for overall morbidity has not been assessed previously in relation to ASD II morbidity. We did not include PPHN in our regression analyses, as it may be classified as a mediator of morbidity in children born preterm and is linked to premature birth as well as to Down syndrome^12,24,25^. Altered pulmonary vascular resistance and delayed normalization of the pulmonary circulation, high pulmonary pressure, along with the intolerance to pulmonary overflow, can indicate that there might be a need of cardiac drug treatment in children with ASDII and maybe especially those with Down syndrome^26^. We choose to define cardiac morbidity by an increased number of outpatient visits to a pediatric cardiologist and not to a general pediatrician. We argue that children with ASD II and Down syndrome may need more careful cardiac monitoring.

In contrast to our hypothesis, being born preterm was not an independent risk factor for cardiac morbidity. An increasing number of studies indicates children born preterm are at risk of an early ASD II closure and of heart failure and pulmonary hypertension throughout the entire childhood and even during adulthood^12,13,15,27^. Assessing the benefits of early ASD II closure can be difficult, as clinical improvement of ASD II in a preterm-born patient may also be part of a normalization of the pulmonary vascular resistance as a child grows^28^. Premature birth was not associated with an increased risk of cardiac morbidity in our study, which contrasts with results from other studies^12,29^. We believe that the number of children lost to follow up is limited in our study and cannot explain these difference. An intervention was made in 16% of the term born children and 18% of the preterm born children (*p*-value: 0.08) and further studies on the risk of an early intervention in children born preterm is needed. Pre-interventional ASD II-associated morbidity, shown by others, may be associated with other, yet unknown factors.

The number of outpatient visits has previously been used in other studies to assess health care resource utilization and as a measure of morbidity^30^. Setting a cut-off number of outpatient visits to identify morbidity is sensitive, as a low number can hamper the analysis and the interpretation of the results. A low value may introduce low specificity while a high value may result in low sensitivity^21^. We used the third interquartile range of outpatient visits as a cut-off proxy measurement of isolated ASD II morbidity^21^. However, more than five visits may be influenced other factors, such as local traditions. By the national approach we believe this study has ruled out the risk of a such skewed outcome measures.

ASD II diagnosis may be confused with PFO and it is of great importance to separate these diagnosis when assessing morbidity in children with ASD II. The SWEDCON register is based on the EPCC coding for diagnosis and will distinguish between ASD (EPCC) 05.04.02) and PFO (EPCC code 05.03.03) diagnosis^19^. All cardiac diagnoses in SWEDCON are set by a pediatric cardiologist or an echo-trained pediatrician and diagnosis in SWEDCON is always based on echo cardiac findings. Thus, the risk of selection bias or misclassification in in this study must be regarded as low. Small ASD II may not be recorded in the SWEDCON register. This must be regarded as equal for all children, and we believe the risk of selection bias due to small ASD II is low. The validity of ASD II diagnoses in SWEDCON has a good coherence between medical records and registered data^31^. Missing information on birth is low, and 98.1% of all births are registered in the Medical Birth Register^32^. This reduces the risk of missing data and increases the validity of retrieved data.

All children with isolated ASD II in Sweden were included in this study. The well documented high national coverage of SWEDCON and the large number of included children strengthens the study. The use of well-known risk factors and a well-defined outcome variable, along with the documented good coherence and validation of data in the registries, are other factors that strengths to the study.

The retrospective approach may limit the study through the risk of selection, recall, and attribution bias. However, by combining two national registries, which both are validated with good agreement with other sources, strengthens the reliability of the collected data and decreases the risk of biases.

The SWEDCON register do not include information type of drug which is a limitation. However, information on drugs used for a clinical condition such as heart failure (the intention to treat) is registered and is used as a proxy for heart failure. Growth retardation, clinical and echo cardiac signs of heart failure, along with frequent infections are known signs of morbidity due to an open ASD II, but not registered in SWEDCON. By using two different proxies to assess ASD II morbidity; medicines for heart failure and number of outpatient visits, we used other ways to assess cardiac morbidity.

## Data Availability

The data that support the findings of this study are available from Socialstyrelsen (https://bestalladata.socialstyrelsen.se/ (Swedish)) and Uppsala Clinical Research Center and SWEDCON (https://www.ucr.uu.se/swedcon/forskning/forskning (Swedish)), but restrictions apply to the availability of these data, which were used under license for the current study, and so are not publicly available.
